# Stereotactic ablative radiotherapy and systemic treatments for extracerebral oligometastases, oligorecurrence, oligopersistence and oligoprogression from lung cancer

**DOI:** 10.1186/s12885-019-6449-8

**Published:** 2019-12-19

**Authors:** Manon Kissel, Isabelle Martel-Lafay, Justine Lequesne, Jean-Christophe Faivre, Cécile Le Péchoux, Dinu Stefan, Victor Barraux, Cédric Loiseau, Jean-Michel Grellard, Serge Danhier, Delphine Lerouge, Christos Chouaid, Radj Gervais, Juliette Thariat

**Affiliations:** 1 Centre de lutte contre le cancer François Baclesse/ ARCHADE, radiotherapy department, 3 avenue du Général Harris, 14000 Caen, France; 2 Institut Gustave Roussy, radiotherapy department, 114 Rue Edouard Vaillant, 94800 Villejuif, France; 3 Centre de lutte contre le cancer Léon Bérard, radiotherapy department, 28 Promenade Léa et Napoléon Bullukian, 69008 Lyon, France; 4 Centre de lutte contre le cancer François Baclesse, clinical research department, 3 avenue du Général Harris, 14000 Caen, France; 5 Institut de Cancérologie de Lorraine, radiotherapy department, 6 Avenue de Bourgogne, 54519 Vandœuvre-lès-Nancy, France; 6 CHI de Créteil, pneumology department, 40 Avenue De Verdun, 94000 Créteil, France

**Keywords:** Lung cancer, Oligometastasis, Stereotactic radiotherapy, Systemic treatment, Survival

## Abstract

**Background:**

Stereotactic irradiation (SBRT) is a standard of care for inoperable stage I lung cancer and brain oligometastases from lung cancer but is controversial for extracranial oligometastases. We assessed outcomes of lung cancer patients with extracranial metastases in oligometastatic, oligorecurrent, oligopersistent and oligoprogressive settings (“oligometastatic spectrum”) under strategies using SBRT +/− systemic treatments.

**Methods:**

A retrospective multicentric study of consecutive lung cancer adult patients with 1–5 extracranial metastases treated with SBRT was conducted.

**Results:**

Of 91 patients (99 metastases, median age 63, 64.8% adenocarcinomas, 19.8% molecular alterations), 11% had oligometastases, 49.5% oligorecurrence, 19.8% oligopersistence and 19.8% oligoprogression. Of 36% of patients under systemic treatments at initiation of SBRT, systemic treatment interruption was performed in 58% of them. With median follow up of 15.3 months, crude local control at irradiated metastases was 91%, while median distant progression-free survival (dPFS) and overall survival were 6.3 and 28.4 months (2-year survival 54%). Initial nodal stage and oligometastatic spectrum were prognostic factors for dPFS; age, initial primary stage and oligometastatic spectrum were prognostic factors for survival on multivariate analysis. Patients with oncogene-addicted tumors more frequently had oligoprogressive disease. Repeat ablative irradiations were preformed in 80% of patients who had oligorelapses. Worst acute toxicities consisted of 5.5% and one late toxic death occurred.

**Conclusion:**

The oligometastatic spectrum is a strong prognosticator in patients undergoing SBRT for extracranial metastases. Median survival was over two years but dPFS was about 6 months. Continuation of systemic therapy in oligoprogressive patients should be investigated.

## Background

Lung cancer is the primary cause of death from cancer among men and second leading cause among women, both in France and worldwide. This is primarily due to propensity for metastases. However, metastatic disease appears to harbor different prognoses that are dependent on tumor bulk and kinetics. In particular, oligometastatic disease describes an intermediate state between local disease and multimetastatic cancer. This change of paradigm and prognosis has been integrated into the 8th lung cancer TNM classification [1]. However, proper estimates of the prevalence of oligometastatic lung cancer patients would require consistent definition of oligometastatic disease, accurate description of disease in databases and clinical trials (which include various metastatic disease bulks) and full diagnostic work up. Rough estimates suggest that single metastasis be present in 7% of patients but drops to only 1% using Positron Emission Tomography (TEP-TDM) [2, 3]. As a consequence, it has been difficult to address the question of a potential benefit of aggressive ablative treatments in oligometastatic lung cancer [4]. Surgical removal of adrenal, cerebral or pulmonary metastases have long been performed for lung cancer patients but may not be appropriate for all metastatic sites and in case of several synchronous metastases. On the other hand, first line platin-based doublet yields low response rates [5]. Similarly, 20% of patients have a targetable genetic alteration and can exhibit dramatic tumor response but acquired resistance is usually unavoidable within about a year. Thus, therapeutic options were limited before the recent rise of immunotherapy. Immunotherapy may however be limited by PD1/PDL1 expression and has only been available since 2017. Altogether, systemic therapies alone may not be optimal in disease settings, such as oligometastatic lung cancer, where long term control can be expected. Recent retrospective data suggest that lung metastases from various primaries may benefit from various combinations of stereotactic ablation and systemic treatments that can be personalized based on disease progression and number of metastases [6]. Recent prospective data also suggest that consolidative stereotactic irradiation improves survival in primarily polymetastatic lung cancers that have been downstaged to oligometastatic stage after chemotherapy [7]. Thus, consistent with the standard role of stereotactic irradiation in inoperable lung primaries and brain oligometastases from lung primaries, the use of stereotactic irradiation in oligometastases (any extracranial site) from lung primaries might provide a survival benefit. Yet, series on extracranial metastases from lung cancer are still rare.

The goal of our retrospective observational multicentric study was to assess practice patterns of stereotactic irradiation and outcomes of consecutive lung cancer patients with limited metastatic disease in the « oligo spectrum ».

## Methods

**Fig. 1:**
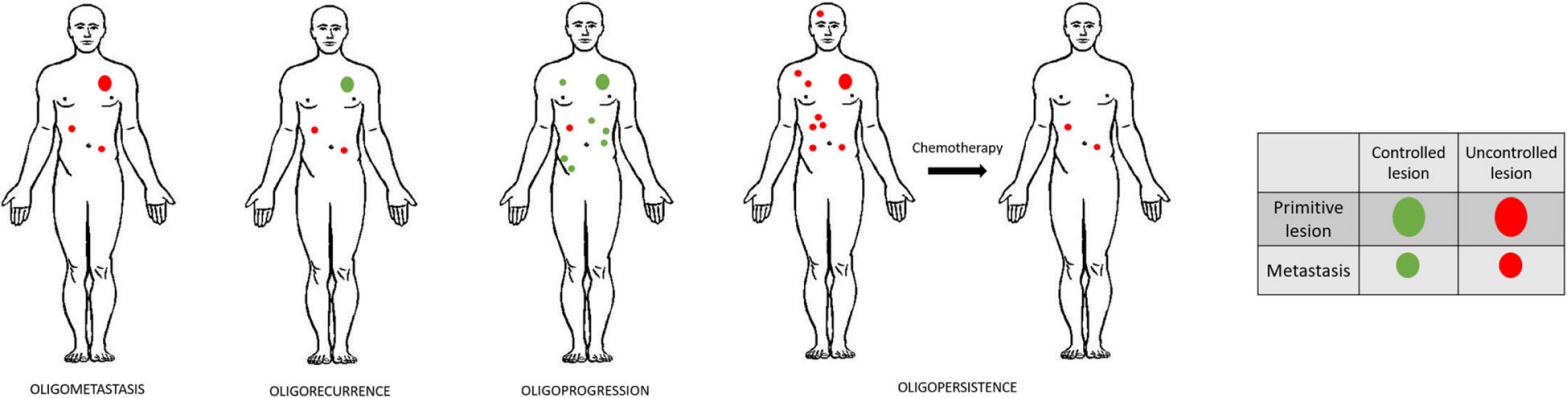
definition of oligometastatic spectrum

This retrospective study was institutional review-board, INDS (Institut National des Données de Santé), CEREES (Comité d’Expert pour les Recherches, les Etudes et les Evaluations dans le domaine de la Santé) and CNIL (Commission Nationale de l’Informatique et des Libertés) -approved. Patients over 18 were included after ablative stereotactic irradiation on all extracranial oligometastatic lesions (one to five) from their lung cancer in the following situations of the oligometastatic spectrum: oligometastases at diagnosis, oligorecurrence defined as oligometastatic relapse after primary, oligopersistence defined as stable residual disease sites after systemic treatment and oligoprogression in a polymetastatic context with progressive lesions while all other lesions are controlled with systemic treatment (Fig. 1) [6, 8]. Stereotactic irradiation had to be performed between January 2012 and August 2016. Patients with cerebral metastases controlled for at least 3 months before extracranial stereotactic irradiation were allowed. Any systemic treatment was allowed.

Clinical evaluation with a radiation oncologist was planned before and after treatment and weekly during the treatment. Surveillance was carried out with clinical exams and regular CT-scans. Therapeutic response evaluation (with central review assessment) was made using RECIST 1.1 criteria. Toxicity severity was reported utilizing CTCAE (Common Terminology Criteria for Adverse Events) scale, version 4.0. Data included treated sites, technical data (doses, fractionation, prescription modalities) and outcomes.

### Statistics

Qualitative variables were described by using numbers and percentages, and quantitative variables by using mean (+/− standard deviation) or median and range in case of non-normal distribution. Overall survival, local and distant recurrence-free survival curves were estimated by the Kaplan-Meier method and calculated from the beginning of stereotactic irradiation. The median time and survival rates at different points since the treatment start were estimated with their 95% confidence interval. The log-rank test and the Cox model were used to compare survival curves according to observed characteristics. The effect of continuous variables on survival was evaluated both continuously and through a log-rank test by dichotomizing the variable either by the median value or by a so-called optimal cut-off value, i.e., the one producing the most significant statistical difference in survival between the two groups thus defined.

## Results

### Population description

Ninety-one (99 treated lesions) patients were included. Patients’ characteristics are reported in (Table 1)**.** Briefly, 64.8% of patients had adenocarcinomas and 19.8% had molecular alterations.

**Table 1:** Patient, tumor and treatment characteristics

	n	%	*N*
	médiane	[min-max]	
Center			91
*CFB*	16	17.6%	
*CLB*	40	44%	
*ICL*	28	30.8%	
*IGR*	7	7.7%	
Age	63.43	[42.39–87.34]	91
Gender			91
*F*	33	36.3%	
*M*	58	63.7%	
PS			90
*0*	39	43.3%	
*1*	43	47.8%	
*2*	8	8.9%	
Smoker (current or former)	73	85.9%	85
Respiratory comorbidity	26	29.2%	89
Cardiovascular comorbidity	24	27.3%	88
Other cancer history	19	21.6%	88
Other significant comorbidity	21	23.9%	88
Histology			91
*Adenocarcinoma*	59	64.8%	
*Squamous cell*	16	17.6%	
*Small Cell Lung Cancer*	8	8.8%	
*Other*	8	8.8%	
Molecular alteration	18	19.8%	91
*EGFR*	8	9%	
*ALK*	2	2.2%	
*KRAS*	4	4.5%	
*HER2*	2	2.3%	
*cMET amplification*	1	1.1%	
*cMET mutation*	1	1.1%	
T			89
*T1*	18	20.2%	
*T2*	35	39.3%	
*T3*	25	28.1%	
*T4*	11	12.4%	
N			89
*N0*	31	34.8%	
*N1*	16	18%	
*N2*	26	29.2%	
*N3*	14	15.7%	
*Nx*	2	2.2%	
Pre-therapeutic PET-TDM	79	87.8%	90
Metastase(s) operability			91
*Yes*	7	7.7%	
*No*	41	45.1%	
*Not reported*	43	47.3%	
Controlled primitive lesion	77	84.6%	91
Number of metastase(s)			91
*1*	83	91.2%	
*> = 2*	8	8.8%	
Characteristics of metastatic evolution			91
*Metachronous*	63	69.2%	
*Synchronous*	28	30.8%	
Indication			91
*Oligopersistance*	18	19.8%	
*Oligometastatic*	10	11%	
*Oligoprogression*	18	19.8%	
*Oligorecurrence*	45	49.5%	
Treated site			99
*Liver*	12	12.1%	
*Lymph node*	7	7.1%	
*Bone*	20	20.2%	
*Lung*	21	21.2%	
*Spine*	12	12.1%	
*Adrenal*	27	27.3%	
*Contralateral to primitive lesion*	13	48.1%	27
*Homolateral to primitive lesion*	14	51.9%	27
Ongoing systemic treatment before irradiation			91
*No*	58	63.7%	
*Yes*	33	36.3%	
Systemic treatment interruption during irradiation			33
*No*	1	2.9%	
*Yes*	32	97.1%	

*CFB* Centre François Baclesse, *CLB* Centre Léon Bérard, *ICL* Institut de Cancérologie de Lorraine, *IGR* Institut Gustave Roussy

Histological verification of at least one metastatic lesion was available in 20% of cases. Among the 8 patients treated for two lesions, one patient was biopsied on both metastatic sites. All other patients were classified as M1a, according to TNM 8th, due to metastatic lymph node or another distant lesion. Overall, 91.2% had a single metastasis. Eleven percent had oligometastases, 49.5% oligorecurrence, 19.8% residual stable disease (for irradiated as consolidative therapy) and 19.8% oligoprogression. Adrenal metastases were present in 27.3% of the patients, lung in 21.2%, bone in 20.2%, liver and spine both in 12.1% and lymph nodes in 7.1%.

Prior other local treatments had been performed in 30% of patients (surgery of primary or metastatic lesion, radiochemotherapy of the primary, radiofrequency, cryotherapy). 35% of the patients never had any systemic treatment. Stereotactic irradiation was performed during first line for 42% of patients, second line for 10% of patients and third line or more for 13% of patients.

### Technical data

Patients were treated with Cyberknife® (Accuray) in the 4 participating centers and some patients (5 cases) with NovalisTx™ (Varian®, Palo Alto, California, USA and BrainLAB AG, Munich, Germany) in one center and with Synergy® (Elekta) in one center (6 cases). Total dose ranged between 15 to 60 Gy in 2 to 8 fractions. Total median dose was 39 Gy for a biological equivalent dose (BED) of 60.5 Gy on tumor (α/β 10). Median dose per fraction was 8 Gy. Prescription schemes were heterogeneous, the most common being 3 × 15 Gy, 5 × 7 Gy and 5 × 8 Gy. For lung lesions, the most frequent scheme was 4 × 12.5 Gy. For liver lesions, the preferred scheme was 3 × 15 Gy. For adrenal metastases, a 5 to 6 fractions of 7 Gy was preferred.

Isodose of prescription was the 80 and 90% with the Cyberknife® or Novalis™, respectively.

Treatment was delivered in 12 days for a single lesion in average, 33 days for two lesions. Mean GTV was 7 cm^3^ and PTV 26 cm^3^.

### Outcomes

Median follow-up was 15.3 months.

#### Toxicity

Worst acute toxicities consisted of 5.5% grade 3, mostly as pain or fatigue. One patient experienced necrosis leading to major pain during adrenal irradiation (GTV 25 cc; 5 × 7 Gy with Cyberknife®), requiring treatment interruption. Another patient with adrenal oligometastasis treated with Novalis™ (PTV 80 cc; 5 × 7.5 Gy) had an abscess, a septicemia, requiring antibiotics and drainage.

Late toxicities consisted of grade 1–2 neuropathic pain, without fracture after spinal radiation in 13% of patients. Grade 1–2 pneumonitis occurred in 8% of patients. No grade 3 or 4 toxicity was noted. Nevertheless, one toxic death occurred following stereotactic irradiation of a sphenoidal lesion (further to osteo-meningeal breach, meningitis, and septic shock).

#### Local response

**Fig. 2:**
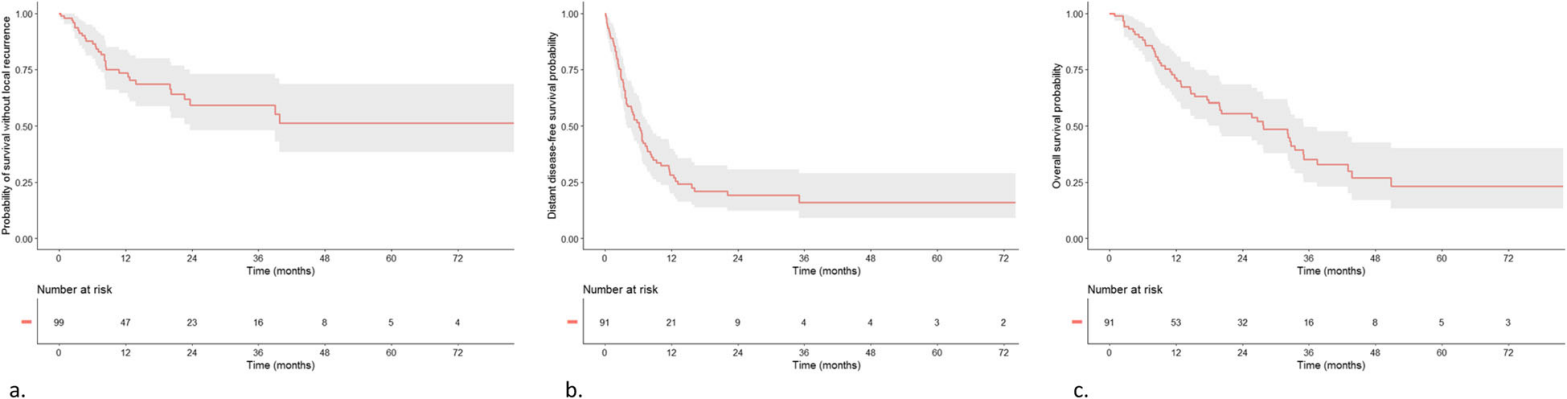
local control (**a**), distant progression-free survival (**b**) and overall survival (**c**)

Best local response was evaluated for each irradiated lesion: objective response rate at irradiated sites was 91% including complete response in 44%, partial response in 27%, stable in 20% and progression in 9% (Fig. 2). Local response was significantly better when GTV was less than 3 cc: *p* = 0.008, HR = 0.263 [0.088; 0.789] and PTV less than 18.5 cc: *p* = 0.008, HR = 0.264 [0.088; 0.795]. Dose, protraction and metastatic site were not associated with local response.

#### Distant progression-free survival

Median distant progression-free survival (dPFS) was 6.3 months [4; 8.1] (Fig. 2). In multivariate analysis, initial nodal status and oligometastatic spectrum (Fig. 1) were predictive of dPFS (Table 2). Median dPFS was 7.8 months for node-negative patients and 3.9 months for node-positive patients: *p* = 0.035; HR = 1.714 [1.012; 2.903]. Oligoprogressive patients had the worst dPFS. Better dPFS was associated with prolonged free interval, with a threshold of 2 years: *p* = 0.001; HR = 2.405 [1.282; 4.509] only on univariate analysis. Among the 68 patients who had out of field relapse, 51% (*N* = 35) had oligorelapses and 35% more than one oligorelapse. Of these, 44% of oligorelapses occurred in the same organ as at first relapse. Overall, 80% of patients had at least more than one local treatment and 70% of the patients underwent ablative treatments for all their subsequent oligorelapses.

**Table 2:** prognostic factors of distant progression free survival and overall survival

	PFS				OS			
	Univariate			Multivariate	Univariate			Multivariate
	HR	IC95	*p*	*p*	HR	IC95	*p*	*p*
PET-TDM			*0.59*		*0.44*	*[0.20,0.94]*	***0.068***	
Oligometastatic site			*0.136*				***0.02***	*0.37*
Indication (oligometastatic spectrum) (ref = oligometastases)			***0.04***	***0.029***			***0.03***	***0.02***
Center			*0.498*				*0.477*	
Age (ref= > 63 y)	*0.69*	*[0.43,1.12]*	*0.134*		*0.42*	*[0.23,0.76]*	***0.004***	***0.003***
Gender (ref = male)	*0.89*	*[0.55,1.46]*	*0.65*		*1.11*	*[0.62,1.98]*	*0.731*	
Smoking habits	*0.66*	*[0.34,1.26]*	*0.226*		*1.25*	*[0.53,2.95]*	*0.593*	
Comorbidities	*0.999*	*[0.61,1.65]*	*0.992*		*1.42*	*[0.78,2.57]*	*0.223*	
Free interval (ref > 516 days)	*2.41*	*[1.28,4.51]*	***0.001***		*3.23*	*[1.37,7.61]*	***0.002***	
Primary cancer controlled	*1.24*	*[0.63,2.44]*	*0.498*		*1.14*	*[0.48,2.70]*	*0.77*	
Number of metastatic lesions =2 (ref = 1)	*1.18*	*[0.53,2.60]*	*0.695*		*0.962*	*[0.38,2.43]*	*0.928*	
Number of previous systemic treatment lines			*0.777*				*0.133*	
Synchronous lesion (ref = metachronous)	*0.90*	*[0.53,1.54]*	*0.716*		*1.09*	*[0.60,1.99]*	*0.779*	
Histology			*0.985*				*0.685*	
EGFR mutation or ALK translocation	*1.56*	*[0.77,3.15]*	*0.252*		*0.83*	*[0.37,1.85]*	*0.581*	
Initial T stage (ref = T1)								
T2	*1.3*	*[0.66,2.58]*	*0.2*		*1.79*	*[0.73,4.4]*	***0.02***	***0.008***
T3	*0.8*	*[0.39,1.75]*			*0.79*	*[0.29,2.2]*		
T4	*1.9*	*[0.84,4.5]*			*3*	*[1.05,8.4]*		
Initial N+ status (ref = N0)	*1.71*	*[1.01,2.90]*	***0.035***	***0.022***	*1.29*	*[0.69,2.425]*	*0.411*	

Boldface entries = statistically significant

#### Overall survival

**Fig. 3:**
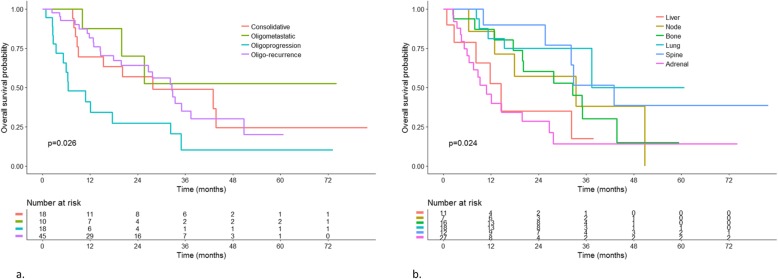
overall survival by oligometastatic spectrum (**a**) and metastatic site (**b**)

Forty-nine patients had died by time of last follow-up, among which 73% of lung cancer-specific death. Median overall survival was 28.2 months [20.07; 35.5]. Overall survival at 6 months, 1 year and 2 years were respectively 88, 71 and 54% (Fig. 2). Oligometastatic spectrum (oligometastatic at diagnosis versus oligoprogressive), initial T stage and younger age were associated with better survival on multivariate analysis. Mean overall survival was 33 months after irradiation of oligorecurrence, 28.2 months after consolidative irradiation and 6.5 months after irradiation of oligoprogression. Median overall survival was not reached in patients with oligometastases at diagnosis (Fig. 3). Metastatic site was associated with survival with patients having spine or lung lesions (versus adrenal or liver lesions) having better survival on univariate analysis (Table 2).

In 18 patients with tumors harboring actionable driver mutations, survival was 34 months [20.4; not reached] versus 27 months [15.5; 43.6] in those without mutations (*p* = 0.581). Patients who had at least one major comorbidity had median survival of 28 months [15.5; 38] versus 33 months [20.1; not reached] in those without comorbidities (*p* = 0.22) (Table 2). For oligorecurrent patients, longer free interval between primary and metastatic spread was associated with better survival, with a threshold of 2 years (*p* = 0.002).

Patients undergoing repeat ablative treatments of their subsequent oligorelapses had a trend for better survival than those with oligorelapses who were not offered ablative treatments (HR = 0.336 [0.114; 0.985] *p* = 0.07).

#### Delaying systemic treatment

At the time of stereotactic irradiation planning, 36% of the patients were under systemic treatment. Among patients undergoing systemic treatment before radiotherapy, 58% could be offered treatment pause (until reprogression) that lasted between 1 to 72 months (mean 8.5 months). Among patients who did not have any systemic treatment at the time of stereotactic irradiation, 88% were treatment-free for 1 to 40 months (mean 11.2 months).

## Conclusion

With 28.2-month overall survival and five-year survival rate of 23%, our results are similar to those of clinical trials involving patients not selected on their response to systemic therapy [9]. In contrast, median distant PFS in our study was 6.3 months i.e. shorter than in these same clinical trial by De Ruysscher et al [9] where median PFS was 12.1 months and 5-year PFS 8%. The difference might be related to inclusion of oligoprogressive patients in our study, these patients having poorer outcomes compared to oligometastases present at initial diagnosis and treated upfront [6]. As for patient / tumor selection, 91% of patients had a single metastatic lesion in this French cohort. This is consistent with recruitment in the phase II trial by De Ruysscher et al and may place these patients in a high range of prognosis based on the number of metastases. This however represents a usual bias on the number of metastases in the selection of oligometastatic patients for ablative treatments [10], despite recent data suggesting a benefit in more advanced disease settings [6]. Recent prospective randomized data in favor of stereotactic ablation are however leading to a progressive switch toward more advanced oligometastatic situations [8, 10, 11]. We did include a large spectrum of oligometastatic diseases [6] to address the various situations encountered in routine practice. We showed that these situations are a relevant prognostic classifier with, from best to worst distant progression-free survival and overall survival, oligometastases at first diagnosis, oligorecurrence, oligoconsolidation and oligoprogression. The oligoprogressive group was marked by a particularly mediocre prognosis, with a 6-months median OS. This group is heterogenous since it included pan-negative patients that had a progressive lesion after a first line of platinum-based chemotherapy but also some oncogene-addicted tumors that progressed slowly after several months of ITK, which probably are very different in terms of tumor phenotype. Patients in this group should be better selected before offering this approach. Because of the low number of patients in this situation in our study one cannot draw conclusions and other studies are needed to identify which patients are on the verge of massive tumor progression and those who indeed have isolated progression. Other prognostic factors for overall survival were age and initial T status. In contrast to Helou et al, our series was exclusively made of metastases from lung primaries. Among patients who did not have any systemic treatment at the time of stereotactic irradiation, 88% were treatment-free for 11.2 months in average, suggesting that in patients with indolent disease, systemic treatment interruption may be proposed. In more aggressive situations of the oligometastastatic spectrum, our study was not specifically designed to investigate the impact of systemic treatment interruption during irradiation. However, similar to the study by Helou et al, systemic treatment could be delayed until reprogression with the use of ablative stereotactic irradiation. Among patients under systemic treatment at initiation of stereotactic irradiation, 58% were offered treatment pauses (until reprogression). Time to reprogression indeed varied between 1 and 72 months and median dPFS was 6.3 months. The short distant PFS in our study might reflect dependence on systemic treatment with rapid distant reprogression after interruption of systemic treatments in some patients. Although treatment interruption has been advocated to postpone acquired clonal resistance and to improve the quality of life, such interruption should be cautious in more advanced situations of the oligometastatic spectrum. In patients with driver mutations (17% in our series) who are likely to be addicted to systemic treatments and in patients with oligoprogressive disease (who had the worst dPFS in our series), it may then be more appropriate to combine systemic treatments with stereotactic irradiation. At the time of stereotactic irradiation planning, 36% of the patients in the current series were under systemic treatment. Toxicity profiles were overall good with 5.5% grade 3 acute toxicity (with no grade 4–5) and one late toxic death but no grade 3–4 toxicity. Severe toxicity (as well as survival, similar to data by Griffioen et al [12]) was particularly present in patients with adrenal metastases. In addition, the literature suggests that association with EGFR inhibitors do not significantly increase toxicity [13]. Radiosensitization with vemurafenib may require more caution although it has been reported as feasible with stereotactic irradiation [14]. Severe toxicities, such as perforation, have repeatedly been reported with stereotactic irradiation in combination with antiangiogenic agents [15]. Waiting 5 half-lives may not be feasible when oncogenic addiction is suspected or potential disease flare-up outside radiation fields is threatening [16]. Thus, alternate systemic, less toxic, treatments and short radiation courses may be proposed in these patients; or omission of ablative radiotherapy may be questioned in patients carrying targetable molecular abnormalities [17]. EGFR mutation carriers were 13.6% in our series versus 11–14% in the literature and ALK rearranged patients 3.4% versus 5% [18]. Mutation status was not predictive of better survival unlike in previous reports [7]. Overall survival was good in this group (34 months) but dPFS was low (4 months) and oligoprogression more common. Consistent with data by Weickhardt et al, continuation in oncogene-addicted tumors should probably be recommended in careful combination with ablative therapy [19].

So, why would stereotactic irradiation provide a benefit in a multidisciplinary strategy? The concept behind it is that of mechanical destruction by irradiation of resistant clones while other disease foci are still controlled by systemic treatments. Due to the lack of randomized trials including a stereotactic arm, the demonstration of the level of evidence for extracranial stereotactic irradiation has lagged behind that of intracranial stereotactic irradiation. Despite criticisms of intrinsically better prognosis and immortal time bias in such cohort studies (selection bias where only survivors until SBRT are included, thus inducing a period of time in the OS analysis where the outcome of the study could not occur), there is accumulating randomized evidence of a survival benefit of adding stereotactic to the treatment of oligometastatic disease in NSCLC. Both phases II of Iyengar et al and Gomez et al were closed early because the control group was considered futile since SBRT as a consolidative treatment after first line chemotherapy in oligometastatic patients almost tripled PFS [7, 20]. SABR COMET multicentric randomized phase II study showed a significant improvement of overall survival in patients treated with SBRT in the oligometastatic or oligorecurrence setting (primary tumor controlled, 18% of lung cancers included), with a median OS of 41 months versus 26 months in the control arm [21]. Phase III studies CORE and SARON are ongoing [22, 23].

While direct comparisons between metastasectomy, stereotactic irradiation or radiofrequency/cryotherapy will unlikely be conducted [24], all options may be equally valid for single peripheral lung metastases. Stereotactic irradiation is probably less invasive and more appropriate for more complex and advanced oligometastatic disease presentations, such as oligoprogression in several synchronous lesions [6]. Repeatability of local ablative treatments is also of interest. Consistent with a study by Salama et al [25], stereotactic irradiation was performed in 80% of oligorelapses in our study, and was associated with better survival than in patients with oligorelapses not undergoing stereotactic irradiation. As ablative irradiation may reduce the duration of prescription of expensive drugs such as targeted therapies, antiangiogenics and immunotherapy, it may even have a positive medico-economic impact [26].

This series has the usual biases of retrospective studies and is of relatively small size. It however identified several prognostic groups in the oligometastatic spectrum, in a homogeneous cohort of lung cancer patients. It suggests that personalization of combined therapies based on oligometastatic pattern, oncogene-addiction is warranted. The oligoprogressive indication seems to be the most challenging. More studies are warranted to help clinicians select the patients in this group that might actually benefit from this approach. Some specific studies addressing the issue of oligo-progressive patients are ongoing, of note randomized phases II studies STOP-NSCLC (NCT02756793) and HALT (NCT03256981).

## Data Availability

The datasets used and/or analysed during the current study are available from the corresponding author on reasonable request.

## Abbreviations

ALK: Anaplastic Lymphoma Kinase
BED: Biological equivalent dose
CEREES: Comité d’Expert pour les Recherches, les Etudes et les Evaluations dans le domaine de la Santé
CNIL: Commission Nationale de l’Informatique et des Libertés
CTCAE: Common Terminology Criteria for Adverse Events
dPFS: Distant Progression-Free Survival
EGFR: Epidermal Growth Factor Receptor
GTV: Gross Tumor Volume
Gy: Gray
HR: Hazard Ratio
INDS: Institut National des Données de Santé
ITK: Inhibitor of Tyrosine Kinase
PDL1: Programmed Death Ligand 1
PFS: Progression Free Survival
PTV: Planning Target Volume
RECIST: Response Evaluation Criteria In Solid Tumours
SBRT: Stereotactic Body Radiation Therapy
TEP-TDM: Positron Emission Tomography - Tomodensitometry
